# The lost stone — Laparoscopic exploration of abscess cavity and retrieval of lost gallstone post cholecystectomy: A case series and review of the literature

**DOI:** 10.1016/j.ijscr.2018.10.020

**Published:** 2018-10-19

**Authors:** Uri Kaplan, Gregory Shpoliansky, Ossama Abu Hatoum, Boaz Kimmel, Doron Kopelman

**Affiliations:** Department of General Surgery B, Emek Medical Center, Afula, Israel

**Keywords:** Abscess cavity, Laparoscopic exploration, Cholecystectomy, Gallstone, Video

## Abstract

**Background:**

Laparoscopic cholecystectomy (LC) is considered the gold standard operation for symptomatic gallstones. Gallbladder perforation occurs in 6–40% of operations. It can lead to spillage of gallstones into the abdominal cavity with possible consequences of long-term complications. We report two cases where a unique use of laparoscopic technique was used to explore abscess cavity and retrieve lost gallstones without penetrating the peritoneal cavity.

**Case presentation:**

We report two cases of peri-hepatic abscess treated with laparoscopic cavity exploration, using 5 mm and 10 mm ports, to retrieve lost gallstones. It was done without entering the peritoneal cavity.

**Discussion:**

Today, minimally invasive technique is used in a variety of surgical cases. We report a novel technique, using laparoscopic skills, to drain abscesses caused by lost gallstones post LC without entering the peritoneal cavity. The use of minimally invasive surgery techniques in order to explore abscess cavities not only help us to extract the cause of the abscess but also prevents another surgery in the abdominal cavity.

**Conclusion:**

Laparoscopic exploration of an abscess cavity is a feasible and safe technique treating long-term complications of gallbladder perforation post LC.

## Highlights

• Gallbladder perforation during laparoscopic cholecystectomy can cause late formation of intra-abdominal abscess.
• Gallbladder perforation should be informed and documented.
• We report a novel technique, using minimally invasive skills, to explore a small cavity of an abscess and retrieve lost gallstones.
• Laparoscopic exploration of the abscess cavity is not only safe and feasible, but enables meticulous exploration of the abscess cavity.

## 1 Introduction

Laparoscopic cholecystectomy (LC) is the gold standard treatment for symptomatic gallstones. One of the common complication of LC, which is less discussed in the literature, is gallbladder perforation. The incidence of gallbladder perforation varies from 1.3% to 40% [1,2]. Gallbladder perforation can cause gallstone spillage and, in most cases, an unsuccessful retrieval of the stones. Most of the spilled stones remain clinically a-symptomatic, however, in 0.04% to 19% of the cases adverse events were reported [2]. Intra-abdominal abscess formation is the most prevalent complication.

Today, the use of minimally invasive technique is growing, and expanded well beyond the traditional surgical cases. In this article, we’ll describe a novel technique to retrieve lost gallstones via laparoscopic exploration of an abscess cavity and review the relevant literature.

The research work has been reported in line with the PROCESS criteria [3].

## 2 Presentation of cases

### 2.1 Case number 1

A 74-year-old male presented to the emergency room (ER) with a six-month vague right upper quadrant (RUQ) pain that was exacerbated during the week prior to his arrival. His past medical history was remarkable for ischemic heart disease, chronic obstructive lung disease, diabetes mellitus hypertension and ten years status-post LC. Radiologic studies confirmed the presence of an abdominal abscess between the liver and the abdominal wall. Under Ultra-Sound (US) guidance, the area of the abscess was marked. The patient was taken to the operating room (OR) for laparoscopic exploration of the abscess cavity by our staff (Video Case no. 1 in Supplementary information). Under general anesthesia the abscess cavity was drained and irrigated using a per-cutaneous drain. A 5-mm port was inserted parallel to the drain and exploration of the abscess cavity revealed bile stones. A 10-mm port was inserted to the abscess cavity parallel to the previous port. The abscess cavity was irrigated and the stones were retrieved using laparoscopic forceps. During the procedure, there was an air leak into the peritoneal cavity which was drained using a veress needle. At the end of the procedure a drain was left in the abscess cavity. The patient received 24 h of prophylactic antibiotics and was discharge home two days post the procedure. During a follow-up meeting at the clinic the drain was removed and he is now 4 years post-surgery symptom free.

### 2.2 Case number 2

A 41-year-old woman was presented to the ER with a one-month vague RUQ pain. Her medical history was remarkable for LC three years before her current admission. Radiologic studies revealed a large abscess close to the liver, adherent to the abdominal wall, and containing two gallstones. An US guided per-cutaneous drain was placed and the patient was scheduled for explorative laparoscopy, by our staff, during the following week, due to technical problems (Video Case no. 2 in Supplementary information). A week later, under general anesthesia, a 5-mm port was inserted to the abscess cavity parallel to the drain. Laparoscopic exploration of the abscess cavity was done using the 5 mm and 10 mm ports. The abscess cavity was irrigated and the gallstones were retrieved. A drain was left in the abscess cavity. The patient was discharged home after 48 h. She returned seven days post the procedure with a clinical and radiological picture of intra-abdominal abscess which was adjacent to the previous one. A per-cutaneous drain was inserted and the patient was discharged home. At the follow-up meeting in the clinic, both drains were removed and the patient remained asymptomatic.

## 3 Discussion

In contrast to open cholecystectomy, where the entire operative field is fully visualized and spilled stone can immediately be retrieved, in the laparoscopic era the chances for misdiagnosis or incomplete retrieval of spilled stones are much higher. Spilled gallstones can lead to numerous long-term complications. Zehetner et al. found, in their review of the literature, 44 different types of complications due to spilled gallstones [4]. Abdominal wall abscess and intra-abdominal abscess were the most frequent. Peritoneal gallstones create an inflammatory process that can lead to partial or complete reabsorption of the stone, abscess formation, granulomatous reaction and even erosion to other abdominal organs [5]. Infected stones, which are more likely to happen in case of pigmented stones, intensify this process.

Unfortunately, perforation of gallbladder during LC and especially, spillage of gallstone, is poorly reported in the operation note. It can cause delay in diagnosis, especially in cases that present several years post operation. Late complication of perforated gallbladder should be considered in any patient who had LC in the past.

The treatment of abscess formation due to lost gallstone necessitate the need for drainage and complete stone removal. The stone can be removed endoscopically, percutaneously or by open surgery. The advantages of the minimally invasive technique over the open one include safe and controlled exploration of the abscess cavity and redundant peritoneal cavity exploration. In review of the literature we found two techniques for abscess cavity exploration that are similar to our technique. The authors used nephroscope [6] and combined percutaneous and a choledocoscope [7]. Both techniques used endoscopic techniques and not laparoscopic technique like we did.

Nowadays, the use of minimally invasive technique, which includes a camera port and working ports, has expanded to different cavities in the human body. We describe a novel technique, which was never described before, using laparoscopic equipment in order to explore the abscess cavity and retrieve the lost gallstone. The use of laparoscopic equipment enables us controlled inflation of the abscess cavity which in turn promotes a meticulous exploration of the abscess cavity for stones and fragments of stones. Our technique, which performed by skilled minimally invasive surgeon, enable a safe and thorough exploration of the abscess cavity. This exploration will extract any fragment of gallstone that could be a nidus for continuous infection.

## 4 Conclusion

Lost gallstone can cause long term complication even several years post-surgery and the documentation can shorten the time for diagnosis. Our novel technique enables meticulous exploration of the abscess cavity using laparoscopic equipment. It adds another treatment option for the minimally invasive surgeon to treat abscess drainage caused by gallstone.

## Conflicts of interest

Uri Kaplan, Gregory Shpoliansky, Ossama Abu Hatoum, Boaz Kimmel and Doron Kopelman have no conflict of interest.

## Funding source

Uri Kaplan, Gregory Shpoliansky, Ossama Abu Hatoum, Boaz Kimmel and Doron Kopelman have no financial ties to disclose.

## Ethical approval

As my institution’s IRB policy states that studies of less than four subjects (my paper has two) are not considered Human Research, my submission is exempt from IRB and Ethics approval.

## Consent

Written informed consent was obtained from the patient for publication of this case report and accompanying images. A copy of the written consent is available for review by the Editor-in-Chief of this journal on request.

## Author contribution

UK designed the report, reviewed the literature, and drafted the manuscript. GS performed the study conception and design. OAH and BK participated in designing the report. DK carried out the surgical procedure and participate in critical revision.

## Registration of research studies

UIN: 4176.

## Guarantor

Uri Kaplan.

## Provenance and peer review

Not commissioned, externally peer reviewed.
